# PCL/Mesoglycan Devices Obtained by Supercritical Foaming and Impregnation

**DOI:** 10.3390/pharmaceutics11120631

**Published:** 2019-11-26

**Authors:** Paola Franco, Raffaella Belvedere, Emanuela Pessolano, Sara Liparoti, Roberto Pantani, Antonello Petrella, Iolanda De Marco

**Affiliations:** 1 Department of Industrial Engineering, University of Salerno, Via Giovanni Paolo II, 132, 84084 Fisciano (SA), Italy; pfranco@unisa.it (P.F.); sliparoti@unisa.it (S.L.); rpantani@unisa.it (R.P.); 2 Department of Pharmacy, University of Salerno, via Giovanni Paolo II, 132, 84084 Fisciano (SA), Italy; rbelvedere@unisa.it (R.B.); epessolano@unisa.it (E.P.)

**Keywords:** mesoglycan, wound healing, supercritical foaming and impregnation, transdermal drug delivery

## Abstract

In this work, a one-shot process for the simultaneous foaming of polycaprolactone (PCL) and impregnation of mesoglycan (MSG) into the porous structure was successfully attempted. Supercritical carbon dioxide plays the role of the foaming agent with respect to PCL and of the solvent with respect to MSG. The main objective is to produce an innovative topical device for application on skin lesions, promoting prolonged pro-resolving effects. The obtained device offers a protective barrier to ensure a favorable and sterilized environment for the wound healing process. The impregnation kinetics revealed that a pressure of 17 MPa, a temperature of 35 °C, and a time of impregnation of 24 h assured a proper foaming of PCL in addition to the impregnation of the maximum amount of MSG; i.e., 0.22 mg_MSG_/mg_PCL_. After a preliminary study conducted on PCL granules used as brought, the MSG impregnation was performed at the optimized process conditions also on a PCL film, produced by compression molding, with the final goal of producing medical patches. Comparing the dissolution profiles in phosphate buffered saline solution (PBS) of pure MSG and MSG impregnated on foamed PCL, it was demonstrated that the release of MSG was significantly prolonged up to 70 times. Next, we performed functional assays of in vitro wound healing, cell invasion, and angiogenesis to evaluate the biological effects of the PCL-derived MSG. Interestingly, we found the ability of this composite system to promote the activation of human keratinocytes, fibroblasts, and endothelial cells, as the main actors of tissue regeneration, confirming what we previously showed for the MSG alone.

## 1. Introduction

After a skin lesion, the wound healing proceeds as a dynamic process that involves several stages, mainly the inflammation, the re-epithelialization, and the tissue remodeling. The skin tissue repair occurs thanks to the interaction of different extracellular matrix (ECM) components, including glycosaminoglycans (GAGs) and proteoglycans (PGs), that play a significant role in each stage of the wound healing process [1]. In this ECM, several cell populations attend to the granulation, re-epithelialization, and tissue remodeling, following the initial haemostatic and inflammatory phases [2]. Keratinocytes, fibroblasts, and endothelial cells are well described as mainly involved in skin tissue regeneration [1,3]. Once activated, keratinocytes migration is followed by proliferation and differentiation; these three events are repeated cyclically to repair the epithelium. After a skin lesion, also fibroblasts become ready to differentiate in myofibroblasts, a key event in the granulation process of tissue repair [4].

In the recent years, new pharmaceutical routes were searched for a proper epithelium repair, but they are still inadequate or even ineffective. On the other hand, mesoglycan (MSG) revealed to be particularly effective in the wound healing [4,5,6,7]. MSG consists in a mixture of GAGs, which are long and linear polysaccharides, extracted from porcine intestinal mucosa. It induces keratinocyte migration and early differentiation, fibroblasts activation, and the organization of the endothelial cells in capillary-like structures [4,5,6,7]. However, long-term therapies based on the use of mesoglycan could be required for a proper re-epithelialization, leading to repeated dosages that can induce side effects on patient’s health. Recently, the attention was focused on the development of new topical devices to minimize the undesired effects, avoiding the first-pass metabolism and high plasma levels generally caused by oral formulations [4,7,8,9]. With the advancement in technology, thousands of products have been developed by targeting various aspects of healing process [10]. Therefore, the development of innovative topical systems for application on wounds, lesions, and chronic skin ulcers still represents a challenge. These medical patches often represent a protective barrier that assure a favorable environment for the wound healing and, at the same time, the tissue formation is promoted thanks to a direct action of the drug [4,7]. A valid answer to troubles associated with long-term therapies can be new wound dressings with a drug controlled release, obtained by using composite particles, nanofibers, hydrogels, film, aerogels, etc. [11,12].

An innovative solvent-free technique to produce polymer/drug composite systems is the impregnation of an active principle into porous polymeric matrices using supercritical carbon dioxide (scCO_2_) [13,14,15]. In this process, the scCO_2_ acts as a solvent, since it solubilizes the drug to be impregnated thanks to its high solvent power, and as a plasticizing and swelling agent for polymer applied as support [14], because scCO_2_ rapidly penetrates into the polymeric matrix. Moreover, post-processes for solvent separation are not necessary, since scCO_2_ is easily removed by depressurization, and it is possible to process also thermosensible compound without degradation, thanks to the scCO_2_ moderate critical conditions (Tc = 31.1 °C, Pc = 7.38 MPa). [16] In some previous studies, one-step procedures for the simultaneous PCL foaming and impregnation via supercritical CO_2_ were developed [17,18,19]. For example, Campardelli et al. [18] optimized the foaming conditions of PCL granules and, then, studied the one-step foaming and impregnation process to obtained medical patches loaded with nimesulide, a non-steroidal anti-inflammatory drug. It was demonstrated that the best foaming of PCL was reached at the operating temperature and pressure of 35 °C and 17 MPa, respectively.

For the attainment of composite systems with a prolonged release, a key role is played by the polymeric carrier that has to be accurately selected. The use of polycaprolactone (PCL) seems to be particularly promising, because of its biocompatibility and its capability to prolong the drug release. In the last decades, compression molding has been one of the most diffused processes for polymeric part production [20,21,22,23]. High automation, high geometrical accuracy, and the fast cycle time, which means high productivity, are the main reasons for the diffusion of this process. In addition, the molding processes are very versatile, thus they are employed for different applications, from the production of packaging to pharmaceutical field, which is a more recent area. Furthermore, the use of solvents is not required, this aspect is very important in the pharmaceutical field, where the reduction of solvent residue in the final product is mandatory. 

The purpose of this work is to develop a composite system PCL/MSG to be applied as topical device on the wounds for a proper re-epithelialization process. A one-step foaming and MSG impregnation process using scCO_2_ at different process times was first applied using PCL in form of granulates as received. Then, aiming at producing topical patches, the optimum operating conditions were used to perform the supercritical foaming and impregnation also on PCL film, prepared by compression molding. Finally, this new system has been tested on cell cultures in order to define its effects. We used human keratinocytes, fibroblasts, and endothelial cells evaluating their activation about the main processes at the base of a correct tissue regeneration. Particularly, we assessed in vitro migration and invasion assays and the formation of capillary-like structures.

## 2. Materials and Methods 

### 2.1. Materials

High molecular weight polycaprolactone (PCL, average Mn ~ 80,000 by GPC) was bought from Sigma-Aldrich (Milan, Italy). Carbon dioxide (CO_2_, purity 99%) was purchased from Morlando Group S.R.L. (Sant’Antimo-NA, Italy). Sodium salt mesoglycan (MSG) was supplied by LDO (Laboratori Derivati Organici spa, Vercelli, Italy); it consists of heparin (40% low molecular weight in the range 6.5–10.5 kDa and 60% less than 12 kDa, sulphurylation degree 2.2–2.6), heparan sulfate (UFH-unfranctioned heparin from 12 kDa up to 40 kDa; sulphurylation degree 2.6), and dermatan sulfate, deriving from epimerization of glucuronic acid of chondroitin sulfate (molecular weight 18–30 kDa, sulphurylation degree 1.3) with a total sulphurylation degree equal to 9.1. The manufacturing of the drug complied with the pharmaceutical quality standard required for active substances of biological origin approved in the EU, including those concerned with viral and bovine spongiform encephalopathy (BSE) safety. It was kindly provided and dissolved in the cell medium at an initial concentration of 1 mg/mL. Water was distilled through a laboratory distiller, supplied by ISECO S.P.A. (Treviolo-BG, Italy).

### 2.2. Solubility and Foaming and Impregnation Tests: Apparatus and Procedures

**Figure 1:**
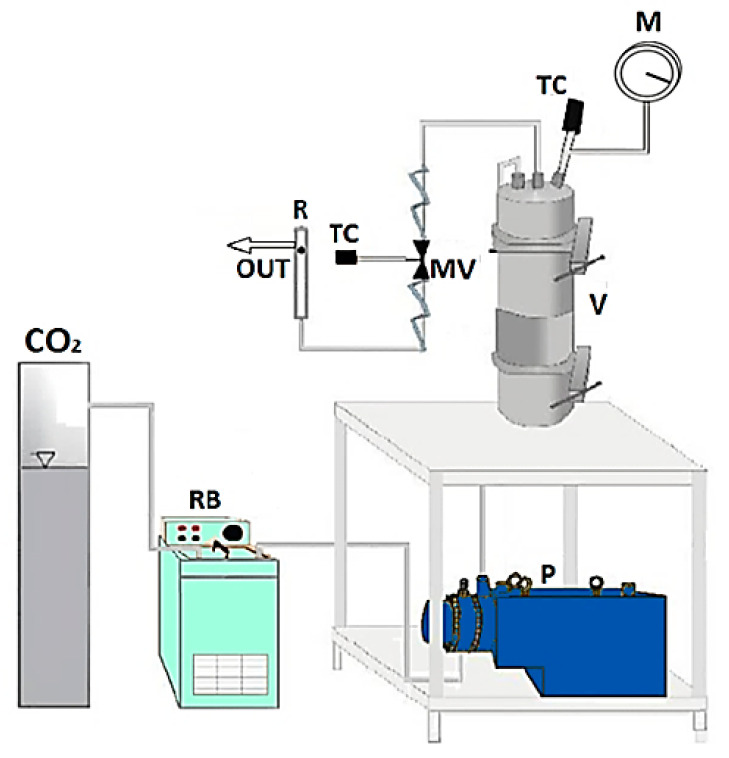
Sketch of the laboratory plant for supercritical foaming and impregnation. CO_2_: supply of carbon dioxide; RB: refrigerating bath; P: pump; V: vessel; MV: micrometric valve; TC: thermocouple; M: manometer; R: rotameter.

The homemade laboratory plant sketched in Figure 1 was used to perform the solubility and foaming and impregnation experiments. Experiments take place in an autoclave; i.e., a stainless steel cylinder (NWA GmbH, Ahlen, Germany) with a 100 mL internal volume, closed on the bottom and on the top with two finger tight clamps. The CO_2_ is cooled before compression thanks to a refrigerating bath and, then, it is fed by a diaphragm piston pump (Milton Roy, mod. Milroyal B, Pont-Saint-Pierre, France). An impeller, which is mounted on the top cap and driven by a variable velocity electric motor, permits a homogeneous mixing in the autoclave. The operating pressure is measured by a digital gauge manometer (Parker, Minneapolis, MN, USA). The thermal control into the autoclave is assured by a proportional–integral–derivative (PID) controller (Watlow, mod. 93, Toledo, OH, USA) connected with electrically controlled thin bands. At the exit of the autoclave, the CO_2_ flow rate is measured by a rotameter. Depressurization is performed by a micrometric valve (Hoke, mod. 1315G4Y, Spartanburg, SC, USA).

The solubility of mesoglycan in scCO_2_ was experimentally determined in the range of pressure 12–17 MPa at a temperature equal to 35 °C, according to a procedure set in previous papers [15,24]. A small stainless steel cylinder containing a weighed amount of MSG was wrapped with paper filter to avoid the drug entrainment and, then, placed on the bottom of the autoclave. Once the autoclave was closed, it was heated up to the desired temperature, whereas the CO_2_ was delivered to reach the desired pressure in the vessel. In order to ensure the equilibrium conditions, the system was stored for 24 h under mechanical stirring. Then, CO_2_ was slowly vented out (about 0.1 MPa/min). At the end of the experiment, the not-dissolved MSG contained in the stainless steel cylinder was weighed, so obtaining the amount of the dissolved MSG by the weight difference. Solubility measurements were repeated in duplicate and the difference between the experiments was less than 3%, probably due to the entrainment of drug through the paper filter during the depressurization.

A static method [24,25] was used to perform the one-step foaming and impregnation tests. The experimental data were determined by charging a weighed amount of MSG (about 20 mg) in a small container opened on the top, in order to allow its contact with scCO_2_, and axially mounted on the impeller. A weighed amount of PCL (about 70 mg) was instead wrapped in a paper filter, to avoid its contact with the solid drug, and placed on the bottom of the autoclave. Finally, the autoclave was closed and the CO_2_ was slowly fed to the vessel, which was heated up to the desired temperature (35 °C). Once the pressure of 17 MPa was reached, the system was stored for a fixed time. Then, the CO_2_ was slowly vented out (constant flow rate of about 0.1 MPa/min) in order to reach the atmospheric conditions and to recover the loaded porous sample from the autoclave. The amount of CO_2_ delivered to the autoclave was determined from the density value, calculated at the operating temperature and pressure by using the Bender equation of state [26]. The quantity of loaded MSG was determined both from the weight increase of the sample after the experiment and by using UV/vis spectroscopy. To prevent that the results are distorted by the residual amount of CO_2_ adsorbed in the sample, the weighs were always carried out two hours after the end of each experiment. Each experiment was performed in duplicate; the difference between the tests was less than 5%, probably due to the possible entrainment of the material through the filter paper during depressurization and to the deposition of non-impregnated MSG on the surface of foamed PCL.

### 2.3. Production of PCL Film by Compression Molding

The pellets of PCL were dried for 2 h under vacuum at a temperature of 27 °C. The films (thickness of 100 µm) were obtained using a compression molding press (Model C, Fred S. Carver Inc., Menomonee Falls, WI, USA), adopting the following process conditions: (a) pre-heating at 120 °C for 5 min, (b) compression-molding at 150 bar for 2 min, and (c) cooling in air. 

### 2.4. Analytical Methods for Samples Characterization

Samples were dispersed on a carbon tab stuck to an aluminum stub (Agar Scientific, Stansted, UK) and analyzed by field emission scanning electron microscopy (FESEM, mod. LEO 1525, Carl Zeiss SMT AG, Oberkochen, Germany). The samples were coated with gold-palladium (layer thickness 250 Å) using a sputter coater (mod. 108 A, Agar Scientific, Stansted, UK), in order to be conductive to obtain the FESEM images.

Fourier transform infrared (FT-IR) spectra were recorded through a M2000 FTIR spectrometer (MIDAC Co., Costa Mesa, CA, USA). Working with a resolution of 0.5 cm^−1^, 16 scan signals were averaged to reduce the noise, in a wavenumber range from 4000 to 450 cm^−1^. The samples were crushed and well mixed with potassium bromide (KBr), which was used as infrared transparent matrix. Discs were obtained by compressing this mixture through a hydraulic press.

A differential scanning calorimeter (DSC, mod. TC11, Mettler-Toledo, Inc., Columbus, OH, USA) allowed us to obtain DSC thermograms by using Mettler STARe system. Approximately 5 mg of samples were heated from 25 to 200 °C with a heating rate equal to 10 °C/min, under a nitrogen atmosphere (50 mL/min).

Drug loadings and dissolution tests were performed using an UV/vis spectrophotometer (model Cary 50, Varian, Palo Alto, CA, USA) at a wavelength of 206 nm. MSG loadings were evaluated by placing each sample in a filter and, then, incubated in 250 mL of phosphate buffered saline solution (PBS) at slightly basic pH equal to 7.4; the system was continuously stirred at 150 rpm and 37 °C. The values of the absorbance were directly measured by the instrument each 0.3 min from time zero up to 100 min, every min in the range 101–1500 min, then every 5 min until the plateau value was reached. Drug loadings were determined considering the absorbance measured at the end of the release profiles; i.e., when all the MSG was released from the foamed PCL to the outer water phase. The absorbance was converted into MSG concentration by using a calibration curve, in order to check the weight increase of the sample measured at the end of the impregnation experiments. Release tests were performed with the same procedure on samples containing an equivalent amount of MSG of 20 mg, in order to do a proper comparison between the unprocessed MSG and MSG impregnated on foamed PCL. The dissolution profile, reported in this paper as the percentage of released MSG vs. the time, represents the average of three analyses.

### 2.5. Cell Cultures

Cell lines were cultured as reported in Belvedere et al., 2017 and Belvedere et al., 2018 [4,7]. Briefly, HaCaT cell line (human immortalized keratinocytes) was purchased from CLS Cell Lines Service GmbH (Eppelheim, Germany) and was maintained in Dulbecco’s modified Eagle’s medium (DMEM) with 10% fetal bovine serum (FBS). BJ cell line (human immortalized fibroblast) was purchased from ATCC (ATCC^®^ CRL2522™, Manassas, VA, USA) and cultured in Eagle’s Minimum Essential Medium (MEM) with 10% FBS, 1% l-glutamine, 1% Sodium Pyruvate, and 1% Non-Essential Amino Acid (NEAA). All the media were supplemented with antibiotics (10000 U/mL penicillin and 10 mg/mL streptomycin). Human Umbilical Vein Endothelial Cells (HUVEC) cell line (ATCC^®^ PCS-100-010™) was maintained in endothelial growth medium (EGM-2), which contains EBM-2 medium (serum free, growth-factor free) supplemented with 2% fetal bovine serum (FBS), human fibroblast growth factor-B (hFGF-B), human epidermal growth factor (hEGF), human vascular endothelial cell growth factor (hVEGF), long R insulin-like growth factor-1 (R3-IGF-1), ascorbic acid, hydrocortisone, and heparin (Lonza). The cells were cultured until passage 10. Then, the cells were stained at 37 °C in 5% CO_2_ –95% air humidified atmosphere and were serially passed at 70–80% confluence.

Powder of sodium MSG was dissolved in cell medium at an initial concentration of 1 mg/mL. Additionally, PBS buffer in which MSG has been dissolved from PCL films was filtered by sterile syringe 0.22 µm filters (Sigma Aldrich, Milan, Italy) and administered to cells. 

### 2.6. In Vitro Wound-Healing 

HaCaT, BJ, and HUVEC cells were seeded in a 24-well plastic plate at 5 × 10^5^, 15 × 10^5^ and 10 × 10^5^ cells, respectively, per well. After 24 h incubation, cells reached 100% confluency and a wound was produced at the center of the monolayer by gently scraping the cells with a sterile plastic p10 pipette tip to create a wound area of about 500 µm. After removing incubation medium and washing with PBS, cell cultures were incubated in the presence of powdered sodium MSG at 50µg/mL and MSG dissolved from PCL film, and harvested at 24, 48, and 72 h, all of them at a final concentration of 50 µg/mL, and in growth medium as control. Both powdered MSG and MSG deriving from PCL were sterilized by sterilizing filtration, through 0.22µm syringe filters. All experimental points were further treated with mitomycin C (10 μg/mL, Sigma Aldrich) to ensure the block of mitosis. The wounded cells were then incubated at 37 °C in a humidified and equilibrated (5% *v/v* CO_2_) incubation chamber of an Integrated Live Cell Workstation Leica AF-6000 LX. A 10× phase contrast objective was used to record cell movements with a frequency of acquisition of 10 min on at least 5 different positions for each experimental condition. The migration rate of individual cells was determined by measuring the wound closure from the initial time (0 h) to the selected time-points (24 h) (bar of distance tool, ASF software, Leica, Wetzlar, Germany). For each wound five different positions were registered, and for each position ten different cells were randomly selected. Carrying on with the images taken every 10 min, the made distance was recorded each 2 h.

### 2.7. Invasion Assay

Cell invasiveness was analyzed as reported in Belvedere et al., 2014 [27]. Briefly, the upper front of trans-well Cell Culture (12 mm diameter, 8.0-fim pore size; Corning Incorporated) was coated with Matrigel (Becton Dickinson Labware), diluted with 3 volumes of serum-free medium and stored at 37 °C until its gelation. Cells were plated in 350 µL of medium serum-free at a number of 4 × 10^4^/insert in the upper chamber of the trans-well. Then, 1.4 mL of supplemented growth medium with or without sodium MSG or PCL-derived MSG at 24, 48, and 72 h were put in the lower chamber and the trans-well was left for 24 h at 37 °C in 5% CO_2_-95% air humidified atmosphere. After that, the medium was aspirated, the filters were washed twice with PBS 1× and fixed with 4% p-formaldehyde for 10 min, then with 100% methanol for 20 min. The filters so fixed, were stained with 0.5% crystal violet prepared from stock crystal violet (powder, Merck Chemicals, Darmstadt, Germany) by distilled water and 20% methanol for 15 min. After that, the filters were washed again in PBS 1× and cleaned with a cotton bud. The number of migrated cells to the lower surface was counted in twelve random fields using an EVOS light microscope (10×) (Life technologies Corporation, Carlsbad, CA, USA).

### 2.8. Tube Formation Assay

A 24-well plate was coated with Matrigel (Becton Dickinson Labware, Franklin Lakes, NJ, USA) mixed to EGM-2 1:1 on ice and incubated at 37 °C for 30 min to allow gelation to occur, as reported in Pessolano et al., 2018 [28]. HUVEC cells were added to the top of the gel at a density of 2 × 10^4^ cells/well in the presence or not of sodium MSG and PCL-derived MSG. Cells were incubated at 37 °C with 5% CO_2_. After 12 h, pictures were captured using EVOS light microscope (10×) (Life technologies Corporation, Carlsbad, CA, USA). The length of each tube was measured and the number of branches was calculated using ImageJ (NIH) (Angiogenesis Analyzer for ImageJ, Madison, WI, USA) software.

## 3. Results and Discussion

The experimental results can be divided in four main steps:

1. Study of the solubility of MSG in scCO_2_;
2. One-step supercritical foaming and impregnation process, to know the time necessary for the equilibrium conditions;
3. Development of MSG-loaded patches by using PCL film produced by compression molding as support for the impregnation at the optimized conditions;
4. Characterization of samples with different analytical techniques.

### 3.1. Solubility Measurements

The solubility of MSG in scCO_2_ was determined at different pressures (12, 15 and 17 MPa) and 35 °C, since this temperature is the best one for the PCL foaming using scCO_2_ [18]. Experimental data were reported in Table 1. It can be noted that the solubility of MSG in scCO_2_ increased by pressure and it seems to reach a plateau value between 15 and 17 MPa. The preliminary studies of the solubility were essential to deduce that is possible to perform the MSG impregnation at the temperature and pressure conditions optimized in the previous work, focused on the PCL foaming; i.e., 35 °C and 17 MPa [18].

### 3.2. Foaming and Impregnation in PCL Granules

**Figure 2:**
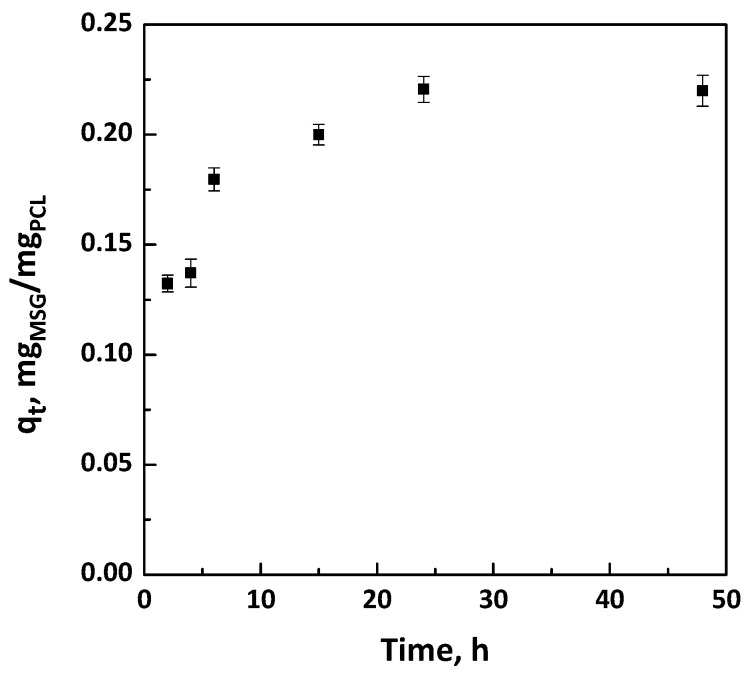
Kinetic curves at 17 MPa and 35 °C for the impregnation of mesoglycan (MSG) on polycaprolactone (PCL) foams.

**Figure 3:**
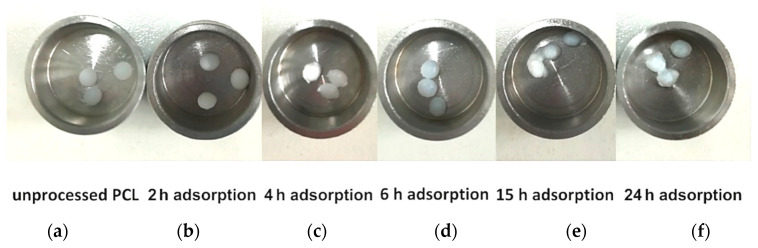
Photographs of (**a**) unprocessed polymer, and impregnated samples at 17 MPa and 35 °C for various contact time: (**b**) 2 h; (**c**) 4 h; (**d**) 6 h; (**e**) 15 h; (**f**) 24 h.

Firstly, the one step foaming and impregnation experiments were performed using PCL granules used as bought without pre-treatment. The impregnation kinetics were determined to know the time required for the complete impregnation of MSG in the PCL foam; i.e., to reach the maximum amount of loaded drug. The uptake was expressed as q_t_; i.e., mg of impregnated MSG per mg of foamed PCL. The experimental data were obtained at 17 MPa and 35 °C, varying the contact time between the polymeric support and the MSG dissolved in scCO_2_ from 2 to 48 h. The impregnation kinetic data were reported in Figure 2. The obtained curves showed that the quantity of loaded MSG increased by increasing the time of impregnation, up to a maximum value of about 0.22 mg_MSG_/mg_PCL_ reached after 24 h. Moreover, different images of PCL granules after the supercritical impregnation at various times investigated (in the range 2–24 h) were also reported in Figure 3. It can be observed that the best foaming of PCL granules occurred after 24 h of impregnation.

It is worth noting that the amount of MSG impregnated in the foamed PCL assures an adequate dosage, overcoming the limits of conventional wound care systems [29]. This outcome confirms the potential of supercritical impregnation to overcome the drawbacks of the traditional technologies in which liquid organic solvents are used; i.e., low drug loadings on/in polymeric matrices [14]. Indeed, when a liquid solvent is used to dissolve and impregnate a drug, its penetration in polymeric support is limited not only by the geometry/surface properties of the polymer matrices (e.g., pore size), but also by the liquid bulk properties (e.g., surface tension of the liquid) [30]. Thanks to the blowing effect and to the quasi-zero surface tension of scCO_2_, an appropriate incorporation of the active principle was reached [14].

In order to check if the experimental and theoretical loadings are in good agreement, the experimental data of the kinetic curves were fitted both with pseudo-first-order and pseudo-second-order models, which are obtained from the integration of the following Equation (1) [31]:

(1) $$\frac{d\left(1-\frac{q_{t}}{q_{e}}\right)}{dt}=-k{\left(1-\frac{q_{t}}{q_{e}}\right)}^{n}$$

where *q_t_* is the amount of MSG impregnated at time “t” (mmol/g) and *q_e_* is the impregnation capacity at the equilibrium (mmol/g).

Impregnation rate constants obtained from the pseudo-first-order and pseudo-second-order kinetics are reported in Table 2. 

It is possible to note that the pseudo-first-order kinetic did not fit the experimental data properly, since the value of R^2^ is far from unity, as shown in Table 1. On the contrary, the pseudo-second-order model fitted the kinetic data correctly, because R^2^ is very close to unity, meaning that the experimental and theoretical loadings were in good agreement.

The pseudo-second-order model [31] was applied in the form:

(2) $$\frac{t}{q_{t}}=\frac{t}{q_{e}}+\frac{1}{k_{2}{q_{e}}^{2}}$$

where *k*_2_ is the pseudo-second-order rate constant (g/mmol h).

**Figure 4:**
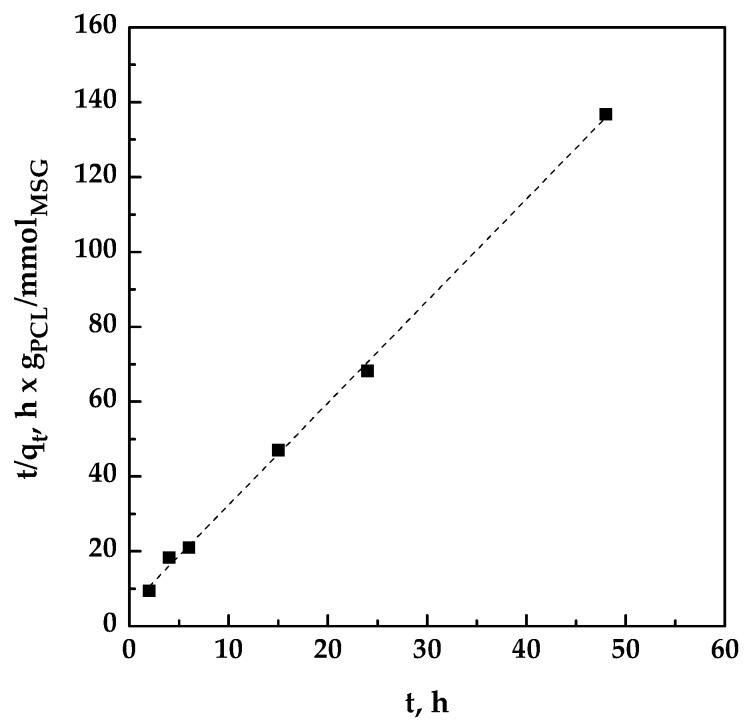
Pseudo-second-order kinetics for the impregnation of MSG on PCL at 17 MPa and 35 °C.

The plot of *t*/*q_t_* versus t was reported in Figure 4; *q_e_* and *k*_2_ were determined from slope and intercept of the fitting.

### 3.3. Foaming and Impregnation in PCL Film

**Figure 5:**
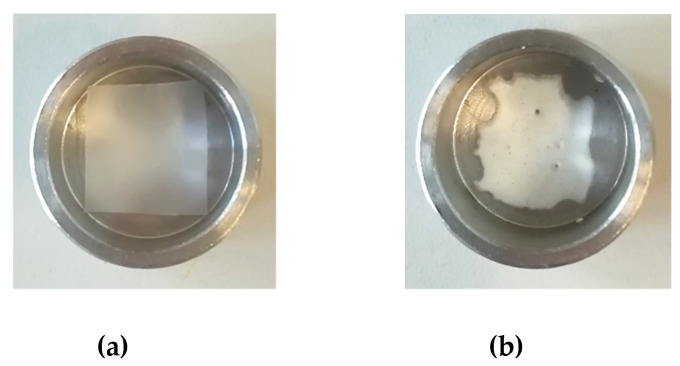
Unprocessed PCL film (**a**), and foamed PCL film after MSG impregnation (**b**).

PCL film with a thickness of 100 µm were produced by compression molding in order to develop topical patches. The supercritical foaming and impregnation of MSG in PCL film was performed at the conditions optimized in Section 3.2.; i.e., 35 °C, 17 MPa and 24 h. Small film pieces of 1.5 cm × 1.5 cm as dimensions were used to conduct the experiments (Figure 5a). At the end of the experiment, a drug loading of about 0.23 mg_MSG_/mg_PCL_ was reached; this value was similar to the MSG loading previously obtained using PCL granules. The aspect of the PCL film foamed and loaded with MSG is reported in Figure 5b. 

**Figure 6:**
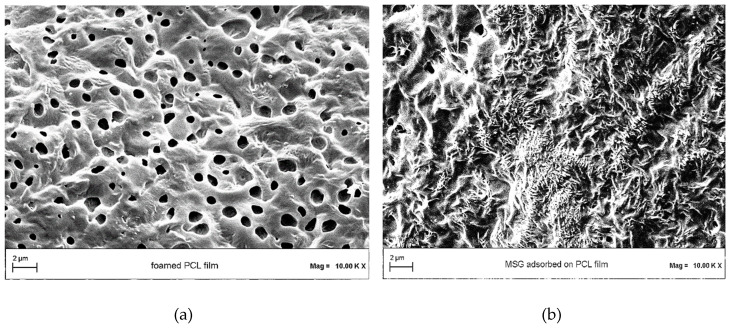
Field emission scanning electron microscopy (FESEM) images of (**a**) PCL film foamed by scCO_2_, and (**b**) MSG impregnated into/on foamed PCL film.

The morphology of foamed PCL was observed by FESEM analysis in absence (Figure 6a) and in presence (Figure 6b) of impregnated MSG. Foamed PCL film is characterized by a porous structure, as highlighted in Figure 6a. After the MSG impregnation, the crystalline drug in form of small needles filled and covered the porosities, as shown in Figure 6b.

Unlike traditional dressings, the use of porous foams as topical formulations provides a proper moist wound environment, in addition to other advantages such as good thermal insulation, better absorption of the exudate, cushioning provided by the porous structure and also a comfortable movement of the injured body part thanks to the flexibility of this material [29,32].

**Figure 7:**
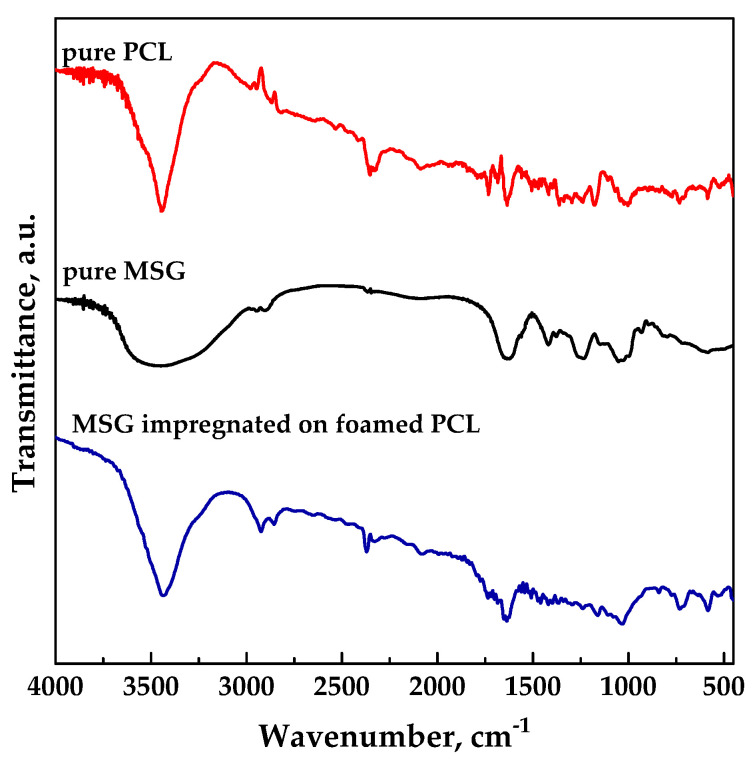
FT-IR spectra for pure MSG and PCL, MSG impregnated on foamed PCL.

FT-IR analyses were performed in order to verify the presence of drug into impregnated samples at the level of functional groups. FTIR spectra of unprocessed MSG, and unprocessed PCL and MSG-loaded PCL film (17 MPa, 35 °C, and 24 h) are reported in Figure 7. Spectra of impregnated sample exhibited the characteristic absorption bands both of the polymer and the drug, confirming the presence of MSG in foamed PCL.

**Figure 8:**
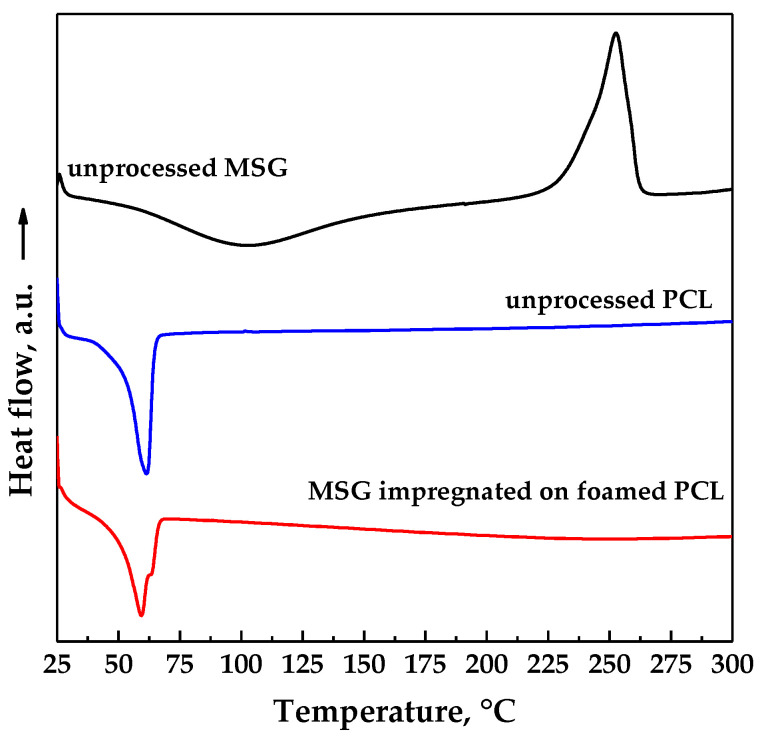
Differential scanning calorimeter (DSC) thermograms of pure MSG and PCL, MSG impregnated on foamed PCL.

DSC thermograms of unprocessed MSG, unprocessed PCL and loaded PCL film (17 MPa and 35 °C for a time of impregnation of 24 h) are reported in Figure 8. MSG curve exhibited an endothermic peak due to the dehydration, followed by an exothermic peak at about 253 °C due to the crystallization. The thermogram of unprocessed PCL exhibited an endothermic melting peak at about 61 °C [33], which shifted at low temperature (around 59.4 °C) for impregnated sample due to the plasticizing effect of supercritical CO_2_ [14,34]. MSG impregnated on foamed PCL showed a thermal behavior similar to the polymer one; this result could be ascribed to the large presence of the polymer with respect to the drug.

**Figure 9:**
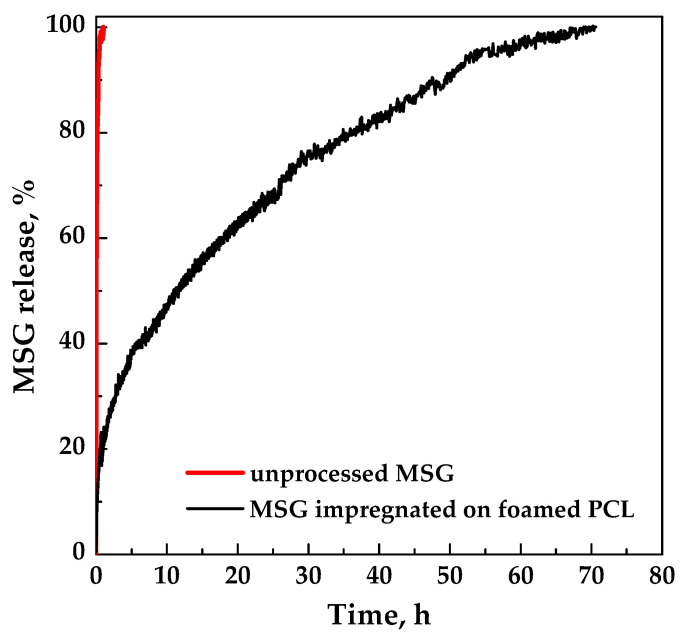
Dissolution tests of MSG in PBS at pH 7.4 and 37 °C.

The dissolution profiles in PBS at pH 7.4 of unprocessed MSG and MSG impregnated on foamed PCL film (17 MPa, 35 °C, and 24 h) are compared in Figure 9. Pure MSG completely dissolved in less than an hour, whereas MSG impregnated on foamed PCL film took about 70 h (about 3 days). A burst-like effect (i.e., the dissolution of drug on/near the surface of the polymeric foam) of about 10% was observed. The comparison of the dissolution profiles demonstrated that the impregnation of MSG into foamed PCL allowed to reach a prolonged release, delaying the complete dissolution of MSG up to about 70 times. Therefore, MSG/PCL system produced could offer a prolonged action on the wound for a proper regeneration of the epithelium. From a clinical point of view, the achievement of a MSG controlled release is an important goal that allows to overcome the major problem of the conventional wound care systems; i.e., the short residence times on the wound site [32]. Indeed, the wound healing process can be improved by a controlled-release delivery that assures a continuous treatment for a long period of time.

### 3.4. Mechanism of Impregnation

Different mechanisms can control the impregnation process; three steps are generally considered [35]:

- Film diffusion, linked to the transport of the solute to the outer surface of the adsorbent;
- Intraparticle or pore diffusion, related to the transport of the adsorbate within the adsorbent pores;
- Release of the adsorbate on adsorbent active sites.

The last step is commonly very fast; therefore its resistance can be neglected [36]. When the support is a polymeric matrix, the impregnation of the drug is often governed by film diffusion at the beginning, followed by pore diffusion [37]. According to the Weber and Morris approach [38], the pore diffusion coefficient *K_id_* can be defined by the following Equation (3):

(3) $$q_{t}=K_{id\;}t^{0.5}+C$$

**Figure 10:**
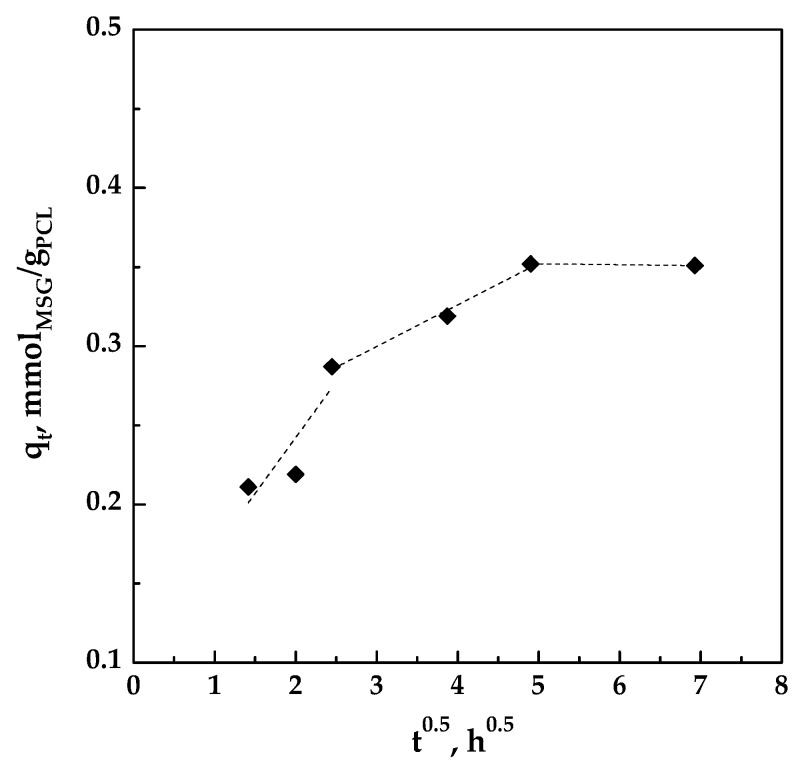
Pore diffusion plot for the impregnation of MSG on foamed PCL.

If *C* = 0 and *K_id_* is constant, *q_t_* is a straight line with intercept equal to zero, and the pore diffusion is the only limiting step; on the contrary, also the film diffusion has to be considered. Plotting *q_t_* versus *t*^0.5^ as shown in Figure 10, a multi-linearity was observed and the intercept of each straight line was not equal to zero; these results indicated that different steps occurred in the impregnation process [36,38,39].

The slopes of the different lines, representing the different stages, are reported in Table 3. It can be observed that K_1d_ > K_2d_ > K_3d_, indicating that the impregnation is controlled by film diffusion at the beginning and that MSG was impregnated on the outer surface of the foamed PCL. At this initial stage, the impregnation rate is fast because the polymeric matrix is unloaded. Then, the MSG is also impregnated on the surface of the inner porosities of the foamed PCL, therefore the process is controlled by pore diffusion [39]. In this step, the diffusion rate decreased due to an increase in diffusion resistance. As the amount of MSG in scCO_2_ decreases, the diffusion rate became lower and the process tends towards the final equilibrium stage, as demonstrated by the very little value of K_3d_.

### 3.5. Effects of MSG Released by Foamed PCL Film on Cells

**Figure 11:**
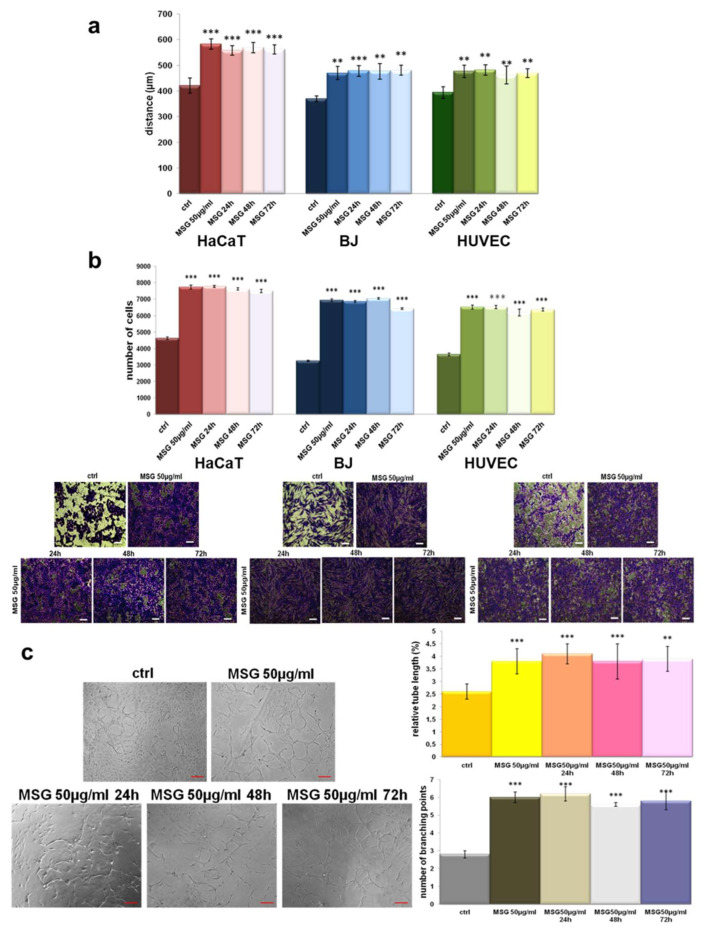
(**a**) Histograms representing the analysis of in vitro wound-healing assay on HaCaT, BJ, and HUVEC cells treated or not with pure MSG, and PCL-derived MSG harvested at 24, 48, and 72 h, all of them at a final concentration of 50 µg/mL. (**b**) Invasion assay on the same cell lines with the relative representative images. Bar = 150 µm. (**c**) Representative images of tube formation by HUVEC cells seeded for 12 h on matrigel: EBM-2 1:1 and treated or not with pure MSG, and PCL-derived MSG. Analysis of tube length and number of branches calculated by ImageJ (Angiogenesis Analyzer tool) software. Bar = 100 µm. The values reported in the graphs are the mean ± SEM from three independent experiments performed in triplicates. Results appeared significant based on Student’s *t*-test, assuming a 2-tailed distribution and unequal variance. *** *p* < 0.001 and ** *p* < 0.01 vs. not treated cells.

Once we characterized the loading and the release of MSG from PCL film, we investigated its effects about the activation of human keratinocytes, fibroblasts, and endothelial cells as the main cell populations involved in skin wound repair. First, we focused on the evaluation of the migration process as the first moment needed for a correct tissue repair. As shown in Figure 11 (panel A), MSG released by PCL film at 24, 48, and 72 h is able to notably increase the migration speed on HaCaT, BJ, and HUVEC cells. Besides the not treated cells, we used also the pure MSG 50 µg/mL as control which confirmed the positive effects of the compound as previously described. Remarkably, in our previous works, MSG lost its effects starting from 48 h after the administration on cells. In particular, we found that fibroblasts are no more able to proliferate and significantly express the metalloproteinases at 72 h of MSG management. Additionally, keratinocytes only preserve a differentiated phenotype at 48 h of MSG treatment. Moreover, all the functional processes deriving by cell activation are evident limited at 24 h of treatment [4,5,7,40].

In order to further analyze the effects of MSG released by PCL in a prolonged time, we assessed the invasion ability of all cell lines as reported in the Material and Methods Section. As for the migration process, cells acquire the ability to faster invade the coating of matrigel in the presence of MSG released from 24 until 72 h from the PCL film (Figure 11, panel B) in a very similar manner of pure MSG dissolved in cell growth medium. 

During wound healing, angiogenic capillary sprouts invade the extracellular matrix thanks to a dynamic interaction between endothelial cells and the surrounding environment. Therefore, the organization of the these cells into a microvascular network throughout the granulation tissue represent a further key step to a proper skin regeneration [41]. Thus, we focused on the in vitro tubulogenesis using HUVEC cells treated or not with pure 50 µg/mL, and MSG released from foamed PCL films at 24, 28, and 72 h at a final concentration of 50 µg/mL. Panel C of the Figure 11 shows the representative bright field images of cells organized in capillaty-like structures when treated with MSG more than the not treated control. Interestingly, for all the experimental times PCL-derived MSG induced a notable in vitro angiogenesis. This aspect is further corroborated by the analisys of relative tube length and of the number of branching points shown in the related histograms. 

## 4. Conclusions

In this work, a one-step supercritical foaming and impregnation process was successfully applied to obtain MSG and PCL foams. The challenge was to produce composite biopolymer/drug systems to be used as topical patches for the wound healing process. The impregnation kinetics of MSG was studied at the pressure and the temperature that guarantee the foaming of PCL; i.e., 17 MPa and 35 °C. Increasing the impregnation time, it was noted that the amount of loaded MSG increased up to a maximum value equal to 0.22 mg_MSG_/mg_PCL_ reached after 24 h, which time also assured the best foaming of PCL granules. The modelling of kinetic data revealed that the impregnation process was properly fitted by a pseudo-second-order model, demonstrating that the experimental and theoretical loadings were in good agreement. Moreover, it was found that the impregnation process was governed by film diffusion at the beginning of the impregnation process, followed by the pore diffusion that allowed to impregnate MSG not only on the external but also on the inner surface of the foamed PCL. Then, the impregnation of MSG was also performed at the optimized conditions (17 MPa, 35 °C, and 24 h) on PCL film, previously prepared by compression molding, in order to develop topical patches. As demonstrated by dissolution tests of MSG in PBS at pH 7.4, the supercritical impregnation of MSG into foamed PCL film is effective to reach a prolonged release of the drug. Indeed, the MSG dissolution rate was 70 times slower than pure MSG when it was impregnated on foamed PCL film.

The functional assays with MSG released by PCL performed in vitro confirmed the positive effects of this mixture. However, the innovative and notable issue of this work is represented by the analysis of the MSG extended-released from a foamed film. In this case, we showed that MSG keeps its notable biological value in each selected experimental times, from 24 to 72 h, compared with the pure MSG at the same final concentration. These data encourage us for the development of MSG containing topical devices also designed to release the active substances in a controlled-time manner. In this work, the system MSG/foamed PCL film appears very interesting from a pharmaceutical point of view. Indeed, this composite system used as topical device can offer a wound protection and a proper regeneration of the epithelium thanks to a prolonged release of MSG. Furthermore, MSG/foamed PCL film obtained by using supercritical CO_2_ can allow to reduce the frequency of administration in long-term therapies, avoiding high and repeated dosages and the associated side effects.

## Figures and Tables

**Table 1:** Solubility of mesoglycan in scCO_2_ at 35 °C and different pressures.

P (MPa)	Solubility mol_MSG_/mol_CO2_
12	3.297 (± 0.071) × 10^−6^
15	4.048 (± 0.113) × 10^−6^
17	4.053 (± 0.068) × 10^−6^

**Table 2:** Impregnation rate constants obtained from the pseudo-first-order and pseudo-second-order models.

Pseudo-First-Order Kinetics			Pseudo-Second-Order Kinetics		
q_e_, mmol/g	k_1_, 1/h	R^2^	q_e_, mmol/g	k_2_, g/mmol h	R^2^
0.351	0.185	0.740	0.367	1.445	0.999

**Table 3:** Pore diffusion rate parameters.

K_1d_, mmol g^−1^ h^−0.5^	K_2d_, mmol g^−1^ h^−0.5^	K_3d_, mmol g^−1^ h^−0.5^
7.01 × 10^−2^	2.64 × 10^−2^	5.0 × 10^−4^
