# Correction: Franco et al. PCL/Mesoglycan Devices Obtained by Supercritical Foaming and Impregnation. *Pharmaceutics* 2019, *11*, 631

**DOI:** 10.3390/pharmaceutics18070774

**Published:** 2026-06-25

**Authors:** Paola Franco, Raffaella Belvedere, Emanuela Pessolano, Sara Liparoti, Roberto Pantani, Antonello Petrella, Iolanda De Marco

**Affiliations:** 1 Department of Industrial Engineering, University of Salerno, Via Giovanni Paolo II, 132, 84084 Fisciano, SA, Italy; pfranco@unisa.it (P.F.); sliparoti@unisa.it (S.L.); rpantani@unisa.it (R.P.); 2 Department of Pharmacy, University of Salerno, Via Giovanni Paolo II, 132, 84084 Fisciano, SA, Italy; rbelvedere@unisa.it (R.B.); epessolano@unisa.it (E.P.)

**Error in Figure**

In the original publication [1], there was a mistake in Figure 11b as published. During figure preparation, the HaCaT control image (ctrl) was inadvertently misallocated. The corrected Figure 11b appears below. The authors state that the scientific conclusions are unaffected. This correction was approved by the Academic Editor. The original publication has also been updated.

## Figures and Tables

**Figure 11:**
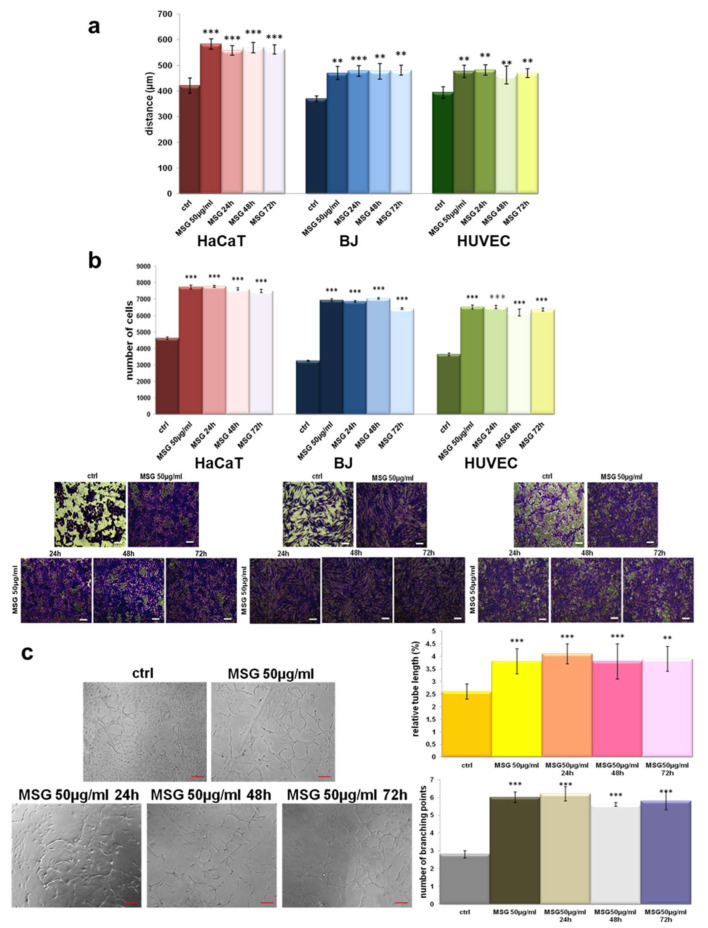
(**a**) Histograms representing the analysis of in vitro wound-healing assay on HaCaT, BJ, and HUVEC cells treated or not with pure MSG, and PCL-derived MSG harvested at 24, 48, and 72 h, all of them at a final concentration of 50 μg/mL. (**b**) Invasion assay on the same cell lines with the relative representative images. Bar = 150 μm. (**c**) Representative images of tube formation by HUVEC cells seeded for 12 h on matrigel: EBM-2 1:1 and treated or not with pure MSG, and PCL-derived MSG. Analysis of tube length and number of branches calculated by ImageJ (Angiogenesis Analyzer tool) software. Bar = 100 μm. The values reported in the graphs are the mean ± SEM from three independent experiments performed in triplicates. Results appeared significant based on Student’s *t*-test, assuming a 2-tailed distribution and unequal variance. *** *p* < 0.001 and ** *p* < 0.01 vs. not treated cells.
